# Supplementary material for: Tryptophan Modulatory Role in European Seabass (Dicentrarchus labrax) Immune Response to Acute Inflammation under Stressful Conditions
Source: Int J Mol Sci. 2022 Oct 18;23(20):12475. doi: 10.3390/ijms232012475 (PMC9604478; doi:10.3390/ijms232012475)
Supplement: Supplementary file 1 [file ijms-23-12475-s001.zip › ijms-1923255-supplementary.pdf]

Supplementary Material

Tryptophan modulatory role in European seabass (*Dicentrarchus labrax*) immune response to acute inflammation under stressful conditions

Marina Machado[1], Diogo Peixoto[1,2,3], Paulo Santos[1], Ana Ricardo[1,2], Inês Duarte[1,2], Inês Carvalho[1,2], Cláudia Aragão[4], Rita Azeredo[1], Benjamín Costas[1,2]

Table S1. Head-kidney gene expression of European seabass reared at high density, fed dietary treatments for 15 days (0h), and sampled 4, 24, 48 and 72 hours post bacterial challenge.

Genes	Dietary treatments CTRL									
	Ø					Stress				
	0h	4h	24h	48h	72h	0h	4h	24h	48h	72h
tgfβ	0.97 ± 0.48 ABC	0.56 ± 0.27 B#	1.01 ± 0.28 AB#	1.38 ± 0.16 A#	0.07 ± 0.02 C	0.70 ± 0.15 BC	1.07 ± 0.22 B*	1.82 ± 0.30 aA*	0.28 ± 0.08 bC*	0.08 ± 0.03 D
tnfα	0.93 ± 0.27 B	2.06 ± 0.51 B	3.05 ± 0.88 bA#	4.68 ± 1.9 A*	0.34 ± 0.11 B	0.74 ± 0.26 C	3.34 ± 1.01 B	5.84 ± 1.52 aA*	0.53 ± 0.20 C#	0.27 ± 0.08 C
cxcr4	1.03 ± 0.31 A	0.80 ± 0.1 A	0.59 ± 0.10 AB	0.71 ± 0.14 B	0.04 ± 0.01 B	0.95 ± 0.15 A	0.79 ± 0.17 B	0.69 ± 0.22 B	0.13 ± 0.02 bB	0.06 ± 0.02 C
il1β	0.94 ± 0.45	224.64 ± 103.91	43.55 ± 17.85	17.71 ± 8.33	1.06 ± 0.41	1.62 ± 0.58	427.65 ± 306.87	60.66 ± 17.11	2.65 ± 0.85	0.90 ± 0.38
cd8α	1.10 ± 0.59 B	1.83 ± 0.62 B	0.89 ± 0.26 C	3.06 ± 0.72 aA*	0.28 ± 0.22 C	0.79 ± 0.23 AB	1.29 ± 0.32 A	1.55 ± 0.64 A	0.36 ± 0.09 bB#	0.21 ± 0.09 B
cd8β	0.90 ± 0.42 B	0.82 ± 0.29 B#	0.65 ± 0.17 B	1.96 ± 0.57 A#	0.28 ± 0.03 B	0.57 ± 0.24 B	1.92 ± 1.27 aA*	1.23 ± 0.61 AB	0.27 ± 0.08 bB*	0.26 ± 0.08 B
cd4	1.22 ± 0.92	36.13 ± 28.26	35.92 ± 27.01 b	5.86 ± 3.15	0.29 ± 0.16	0.94 ± 0.35	28.47 ± 15.60	35.45 ± 19.66	0.61 ± 0.18	0.50 ± 0.14
cd3zeta	1.05 ± 0.37 A	1.20 ± 0.37 A	0.99 ± 0.42 A	1.14 ± 0.25 A*	0.06 ± 0.03 B	0.70 ± 0.16 B	1.33 ± 0.32 A	0.93 ± 0.30 A	0.21 ± 0.07 bB#	0.05 ± 0.02 C
tcrα	1.00 ± 0.17 B	1.14 ± 0.26 B	1.12 ± 0.32 B	2.41 ± 0.89 A*	0.35 ± 0.16 B	0.60 ± 0.21 AB	1.28 ± 0.52 A	1.19 ± 0.13 AB	0.36 ± 0.11 bB#	0.37 ± 0.23 B
mc2r	0.92 ± 0.20	0.90 ± 0.58	0.39 ± 0.21	0.90 ± 0.70	0.05 ± 0.03	0.58 ± 0.27	0.75 ± 0.60	0.27 ± 0.24	0.10 ± 0.04	0.05 ± 0.02
afmid	1.38 ± 1.10 A	0.96 ± 0.36 AB#	1.51 ± 0.51 A	1.98 ± 0.50 A*	0.09 ± 0.03 B	0.51 ± 0.21 BC	2.08 ± 0.58 A*	1.27 ± 0.37 AB	0.44 ± 0.19 bBC#	0.16 ± 0.08 C
ido2	1.00 ± 0.16 C	2.04 ± 0.37 B	2.64 ± 0.98 A	4.60 ± 1.84 A*	0.42 ± 0.19 C	0.44 ± 0.14 B	3.39 ± 0.96 A	3.99 ± 0.97 A	0.74 ± 0.21 bB#	0.39 ± 0.15 B

Genes	Dietary treatments TRP

	Ø					Stress				
	0h	4h	24h	48h	72h	0h	4h	24h	48h	72h
tgfβ	1.10 ± 0.43 B	0.64 ± 0.35 B	1.03 ± 0.48 B	1.24 ± 0.18 B	0.09 ± 0.02 A	0.61 ± 0.16 B	1.12 ± 0.09 AB	0.97 ± 0.13 bAB	1.32 ± 0.30 aA	0.06 ± 0.02 C
tnfα	0.64 ± 0.25 C	1.68 ± 0.70 BC	5.05 ± 2.30 aA	3.65 ± 1.68 AB	0.65 ± 0.46 C	0.79 ± 0.24 B	3.21 ± 1.01 A	3.57 ± 1.23 bA	1.94 ± 0.78 AB	0.24 ± 0.05 B
cxcr4	0.89 ± 0.13 A	0.76 ± 0.11 A	0.69 ± 0.10 A	0.74 ± 0.18 A	0.05 ± 0.02 B	0.80 ± 0.21 A	0.73 ± 0.07 A	0.61 ± 0.19 A	0.85 ± 0.20 aA	0.06 ± 0.03 B
il1β	0.96 ± 0.24	176.78 ± 72.12	77.20 ± 48.06	17.79 ± 7.34	2.24 ± 2.12	1.35 ± 0.72	216.26 ± 65.30	52.94 ± 11.65	9.93 ± 2.59	0.98 ± 0.38
cd8α	0.74 ± 0.23 BC	1.17 ± 0.38 AB	0.82 ± 0.32 B	1.69 ± 0.83 bA	0.33 ± 0.21 C	0.82 ± 0.20 B	1.64 ± 0.31 AB	0.98 ± 0.44 B	1.90 ± 0.65 aA	0.22 ± 0.08 C
cd8β	0.71 ± 0.43 AB	0.92 ± 0.50 AB	0.95 ± 0.36 AB	1.41 ± 0.63 A	0.30 ± 0.08 B	0.60 ± 0.49 AB	0.78 ± 0.18 bAB	1.25 ± 0.70 A	1.41 ± 0.63 aA	0.28 ± 0.08 B
cd4	1.23 ± 0.34 B	35.12 ± 39.77 B	99.78 ± 65.52 aA*	5.89 ± 3.60 B	0.52 ± 0.25 B	1.13 ± 0.39	36.22 ± 29.04	38.83 ± 11.37 #	2.68 ± 1.42	0.47 ± 0.20
cd3zeta	1.01 ± 0.11 B	1.01 ± 0.34 A#	0.98 ± 0.48 A	1.12 ± 0.42 A	0.07 ± 0.02 A	0.71 ± 0.3 B	1.71 ± 0.27 A*	0.77 ± 0.25 B	1.11 ± 0.34 aB	0.05 ± 0.01 C
tcrα	0.88 ± 0.33 BC	0.98 ± 0.34 BC	1.27 ± 0.44 B	2.15 ± 0.64 A	0.40 ± 0.14 C	0.64 ± 0.20 C	1.70 ± 0.51 AB	1.02 ± 0.41 B	1.95 ± 0.76 aA	0.36 ± 0.19 C
mc2r	0.82 ± 0.37	0.81 ± 0.83	1.04 ± 1.12	0.69 ± 0.60	0.04 ± 0.03	0.42 ± 0.20	0.63 ± 0.51	0.55 ± 0.18	0.49 ± 0.30	0.05 ± 0.02
afmid	1.72 ± 1.13 AB	0.67 ± 0.25 BC#	1.20 ± 0.42 ABC	2.05 ± 0.69 A	0.13 ± 0.03 C	0.72 ± 0.24 BC	1.92 ± 0.68 AB*	1.09 ± 0.29 B	2.26 ± 0.73 aA	0.10 ± 0.06 C
ido2	0.74 ± 0.42 C	1.65 ± 0.56 B	2.91 ± 1.30 AB	3.34 ± 1.55 A	0.59 ± 0.24 C	0.58 ± 0.23 B	2.62 ± 0.52 A	2.61 ± 0.78 A	3.03 ± 0.90 aA	0.39 ± 0.16 B

Multifactorial ANOVA

	Diet	Stress	Time	Diet x Stress	Diet x Time	Stress x Time	Diet x Stress x Time	Time				
								0h	4h	24h	48h	72h
tgfβ	ns	ns	<0.001	ns	<0.001	<0.001	<0.001					
tnfα	ns	ns	<0.001	ns	ns	<0.001	<0.001					
cxcr4	ns	0.0158	<0.001	ns	<0.001	0.021	<0.001					
il1β	ns	ns	<0.001	ns	0.008	0.017	ns					
cd8α	ns	0.007	<0.001	<0.001	ns	<0.001	<0.001					
cd8β	ns	ns	<0.001	ns	ns	<0.001	<0.001					
cd4	ns	ns	<0.001	ns	0.026	ns	0.031					
cd3zeta	ns	0.038	<0.001	0.006	0.013	<0.001	0.004					
tcrα	0.033	0.002	<0.001	0.003	0.013	<0.001	<0.001					
mc2r	ns	0.001	<0.001	ns	ns	ns	ns	A	A	A	A	B
afmid	ns	ns	<0.001	ns	<0.001	<0.001	0.012					

	Diet x Time									
	CTRL					TRP				
	0h	4h	24h	48h	72h	0h	4h	24h	48h	72h
tgfβ										
tnfα										
cxcr4										
il1β	B	aA	B	B	B	AB	bA	B	B	B
cd8α										
cd8β										
cd4										
cd3zeta										
tcrα										
mc2r										
afmid										

	Stress x Time									
	Ø					Stress				
	0h	4h	24h	48h	72h	0h	4h	24h	48h	72h
tgfβ										
tnfα										
cxcr4										
il1β	B	A#	B	B	B	B	A*	B	B	B
cd8α										
cd8β										
cd4										
cd3zeta										
tcrα										
mc2r										
afmid										

ido2	ns	ns	<0.001	ns	ns	<0.001	<0.001

Values represent means ± SD (n = 10). Different symbols stand for statistically significant differences attributed to stress. Capital letters stand for statistically significant differences attributed to sampling time. Low case letters stand for significant differences attributed to dietary treatment (Multifactorial ANOVA; Tukey post-hoc test; ns: non-significant; $P \leq 0.05$).
